# A small cohort of FRU^M^ and Engrailed-expressing neurons mediate successful copulation in *Drosophila melanogaster*

**DOI:** 10.1186/1471-2202-14-57

**Published:** 2013-05-21

**Authors:** Kristin L Latham, Ying-Show Liu, Barbara J Taylor

**Affiliations:** 1 Department of Zoology, Molecular and Cellular Biology Program, Oregon State University, Corvallis, Oregon 97331-2914, USA; 2 Molecular and Cellular Biology Program, Oregon State University, Corvallis, Oregon 97361, USA; 3 Present address: Biology Department, Western Oregon University, Monmouth, Oregon 97361, USA; 4 Present address: National Health Research Institutes, Zhunan, Miaoli 35053, Taiwan

**Keywords:** Courtship, Copulation, Drosophila, Fruitless, Engrailed, Central nervous system

## Abstract

**Background:**

In *Drosophila*, male flies require the expression of the male-specific Fruitless protein (FRU^M^) within the developing pupal and adult nervous system in order to produce male courtship and copulation behaviors. Recent evidence has shown that specific subsets of FRU^M^ neurons are necessary for particular steps of courtship and copulation. In these neurons, FRU^M^ function has been shown to be important for determining sex-specific neuronal characteristics, such as neurotransmitter profile and morphology.

**Results:**

We identified a small cohort of FRU^M^ interneurons in the brain and ventral nerve cord by their co-expression with the transcription factor Engrailed (En). We used an *En*-*GAL4* driver to express a *fru*^*M*^ RNAi construct in order to selectively deplete FRU^M^ in these En/FRU^M^ co-expressing neurons. In courtship and copulation tests, these males performed male courtship at wild-type levels but were frequently sterile. Sterility was a behavioral phenotype as these *En-fru*^*M*^RNAi males were less able to convert a copulation attempt into a stable copulation, or did not maintain copulation for long enough to transfer sperm and/or seminal fluid.

**Conclusions:**

We have identified a population of interneurons necessary for successful copulation in *Drosophila*. These data confirm a model in which subsets of FRU^M^ neurons participate in independent neuronal circuits necessary for individual steps of male behavior. In addition, we have determined that these neurons in wild-type males have homologues in females and *fru* mutants, with similar placement, projection patterns, and neurochemical profiles.

## Background

The genes that govern behavior and how these genes function to create specific neural circuits that underlie behavior can be addressed in the model organism, *Drosophila melanogaster*, which has both well-documented stereotyped behaviors and a wealth of genetic information available. In *Drosophila,* male reproductive behaviors are dependent primarily on the activity of the *fruitless* (*fru*) and *doublesex (dsx)* genes, outputs directly regulated by the sex determination hierarchy [1-7]. The male-specific functions of *fru* derive from transcripts generated from the distal-most *fru* promoter (P1) [8-11]. In males, but not in females, P1 *fru* transcripts are translated into male-specific FRU proteins (FRU^M^) [8-11]. FRU^M^ proteins are members of the BTB/ZnF (Broad complex-Tramtrack-Bric-a-brac/Zinc Finger) family, likely function as transcription factors, and are expressed in 2000–4000 neurons in the central nervous system (CNS) and a subset of peripheral sensory neurons [10,12-17]. FRU^M^ neurons are distributed throughout the brain, ventral nerve cord, and peripheral nervous system, in regions previously implicated in male courtship behavior [18-23].

Based on the expression pattern of FRU^M^ and the fact that individual steps of courtship and copulation behavior are differentially affected in specific *fru* mutant genotypes, FRU^M^ function fits both necessary and sufficiency criteria as a regulator of the development and function of neurons that participate exclusively in neuronal circuits used during male courtship and copulation behavior [1,3,6]. For example, males lacking FRU^M^ exhibit abnormalities specifically in sexually dimorphic behaviors (for example, [8,9,11,13,24-29]). When paired with females, mutant males with a complete loss of FRU^M^ function do not produce male courtship behaviors, such as courtship song and attempted copulation, however these males do show some male-male orientation and following behaviors, termed chaining [8,11,30]. Males bearing weaker *fru* mutations display courtship, but have reduced fertility including copulation and sperm transfer defects [26,30,31]. Thus, it has been inferred that subsets of FRU^M^ neurons are organized in circuits for the execution of behavioral subroutines, whereas other FRU^M^ neurons act as command neurons exerting more global control over the timing or sequence of male sexual behaviors. Recent studies using enhancer trap GAL4 insertion lines to deplete the expression of FRU^M^ in subsets of neurons support this model of nervous system organization [32-34]. Although neurons with roles in some aspects of courtship behaviors have been identified, the role of most FRU^M^-expressing neurons in male-specific behaviors, including later behaviors like copulation, has not been established.

To determine the role of other FRU^M^ neurons in male reproductive behavior, we identified and characterized a small subset of neurons by their co-expression with Engrailed (En), a homeodomain transcription factor [35,36]. En has well-known functions in patterning the posterior domains of segments and compartments of imaginal discs (reviewed in [35,37]). In addition, En contributes to the identity of eight neuroblasts and their progeny in each gnathal, thoracic and abdominal hemisegment and in the brain (reviewed in [38-42]) En neurons are found within regions of the CNS known to be important for courtship behavior [18,21]. We identified a small cohort of En and FRU^M^ co-expressing neurons distributed in a segmentally restricted pattern in the brain and ventral nerve cord. Depletion of FRU^M^ in these neurons by *En-GAL4*-driven expression of an inhibitory-RNA transgene directed against male-specific *fru* transcripts resulted in males that courted females vigorously but were frequently unable to successfully copulate or maintain copulation long enough to transfer sperm and accessory materials. Thus, these En/FRU^M^ neurons form part of a specific neuronal network involved in copulation behaviors, supporting the model in which behavioral subroutines are directed by particular neuronal circuits.

## Results

### A subset of FRU^M^ neurons is defined by co-expression with En

**Figure 1:**
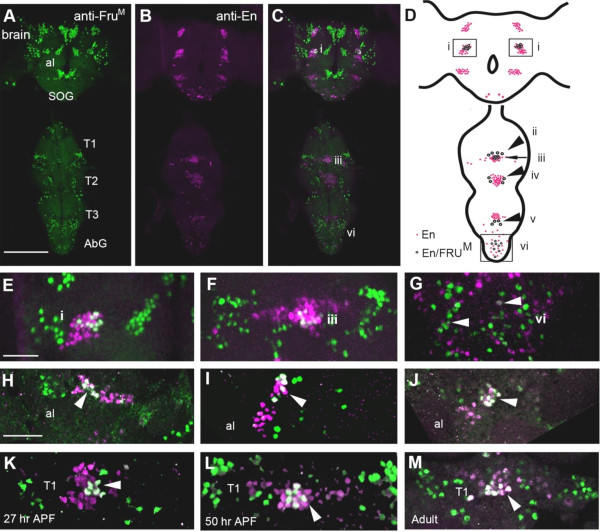
**FRU**^**M **^**and En are co-expressed in a subset of neurons throughout development.** The expression patterns of FRU^M^ and En were analyzed by immunohistochemistry using anti-FRU^M^ (green) and anti-En (magenta) in early pupae through adults and the number of co-expressing neurons counted (Table 1). (**A**-**B**) Doubly-labeled 50 hr APF pupal CNS, anti-FRU^M^ (**A**) and anti-En (**B**) are expressed and co-expressed (**C**) throughout the brain and ventral nerve cord. (**D**) Schematic of En/FRU^M^ co-expression. Pink circles represent En-positive and black-rimmed white circles represent En/FRU^M^ neurons in the brain (box i), T1 midline (arrow, iii), T1-T2-T3 medial cells (arrowheads, ii, iv, v, respectively), and abdominal ganglion (vi). (**E-G**) In optical image overlays of regions i, iii, and vi (noted in panel **D**), En/FRU^M^ co-expression appears white inside cells of the brain (**E**), T1 ganglion (**F**), and abdominal ganglion (**G**). (**H**-**J**) In the anterior mediolateral brain (Box i) from a 27 hr APF pupa (**H**), a 50 hr APF pupa (**I**) and an adult (**J**), En/FRU^M^ are co-expressed (arrowheads) at all stages. (**K**-**M**) In the VNC (arrowheads, ii and iii) from a 27 hr APF pupa (**K**), a 50 hr APF pupa (**L**) and an adult (**M**), FRU^M^/En are co-expressed (arrowheads) in midline neurons of T1 and medial neurons in T1-T3 at all stages. Images are confocal z-stacks through the entire CNS (**A**, **B, C**) or stacks of a subset of z-slices (**E**-**M**). Size bar = 100 μm (**A**) for panels **A** and **B**, 20 μm (**E, H, K**) for panels **E**-**M**.

FRU^M^ neurons contribute to a variety of male-specific reproductive behaviors by their involvement in neuronal circuits mediating courtship and copulation actions. Small groups of FRU^M^ neurons are distributed throughout the brain and ventral nerve cord (VNC; Figure 1A; [8,11,12,15,17,28]). En-positive neurons in the brain are confined to: three compact groups located dorso- medio-, and ventro-laterally within the anterior supraesophageal region, four to five individual neurons near the optic lobes, and four to five clusters of neurons in the subesophageal ganglion (SOG). In the ventral nerve cord, En-positive neurons are found coalesced along the ventral midline in the pro-, meta-, and mesothoracic ganglia (T1, T2 and T3, respectively) and in a large ventral group in the abdominal ganglion (AbG, Figure 1B).

By co-expression of En, we have defined a subset of 53 ± 0.9 FRU^M^ neurons in the brain and VNC, termed En/FRU^M^ neurons (Figure 1C, Table 1; cf. [12,15]). In the brain, En/FRU^M^ co-expressing neurons were found in nearly half of the approximately 50 En-positive neurons in the medial brain En groups (E/F-brain, Figure 1C, D box i, Table 1), thus, 18–22 En/FRU^M^ neurons in total are detected in the brain. In the VNC, about 33 En/FRU^M^ neurons are detected. The greatest number of En/FRU^M^-expressing neurons is found as a compact group of about 14 neurons at the midline of the first thoracic ganglion (E/F-VNC_mid_, Figure 1C, D arrow iii, E, J-L; Table 1). Distinct from the midline group is a set of 3–4 large medial co-labeled neurons in each of the three thoracic ganglia (E/F-VNC_med_, Figure 1C, D arrowheads ii, iv, v). In the abdominal ganglion, there are a small number of co-labeled neurons, usually flanking the midline (E/F-AbG, Figure 1C D box vi, G). Thus, we have identified roughly 53 En/FRU^M^ co-expressing neurons distributed among brain and VNC populations of En neurons.

**Table 1:** **Co-labeled En and Fru**^**M **^**positive neurons in adult male CNS**

**Neuronal region**	**α-En/ α-FruM (male)**
**Brain:**	
E/F-brain	10.0 ± 1.0/side (n = 8)
**Ventral nerve cord:**	
T1 E/F-VNC_mid_	14.0 ± 3.0 (n = 5)
T1 E/F-VNC_med_	3.0 ± 0.3 (n = 5)
T2 E/F-VNC_med_	4.0 ± 0.2 (n = 5)
T3 E/F-VNC_med_	3.0 ± 0.4 (n = 5)
E/F-AbG	9.0 ± 0.4 (n = 5)

The expression of FRU^M^ and En were analyzed by immunohistochemistry using anti-FRU^M^ and anti-En at 50 hr APF, and the number of co-expressing neurons counted. Co-expressing neurons were counted within each section of the central nervous system. Averages ± standard error are reported.

The expression of En developmentally precedes that of FRU^M^ in En/FRU^M^ neurons whether visualized by *fru*-*GAL4* expression pattern or anti-FRU^M^ antibody label. By 8 hours after puparium formation (APF), anti-FRU^M^ antibody labels E/F-brain and E/F-VNC_mid_, and by 12 hours APF the E/F-VNC_med_ and E/F-AbG (data not shown). The number of anti-FRU^M^ neurons labeled from early pupal stages into adulthood is consistent, suggesting that a population of neurons has persistent FRU^M^ expression at levels high enough to be detected by the anti-FRU^M^ antibody (Figure 1H-M). Using a GAL4 driver that recapitulates endogenous FRU^M^ expression, *fruP1-GAL4* driving membrane-bound GFP (UAS*mcd8::GFP,* FBtp0002652) or a nuclear GFP reporter (*UASGFPnls,* FBtp0001204*)* with anti-En staining, we confirm the 53 En/FRU^M^ co-expressing neurons. This pattern is also detected with *fru*^*P1.LexA*^[32] driving expression of GFP (data not shown).

The anti-En antibody recognizes both En and the closely related Invected (Inv) proteins [43-45]. In the embryo, some neurons express only one of these proteins. We assessed whether En/FRU^M^ neurons in the adult expressed both of these proteins by labeling male CNSs from *en*^*Xho2*^*-lacZ* animals and *inv*^*Xba2*^*-lacZ* animals with anti-βgal and anti-FRU^M^. We found complete congruence of FRU^M^ expression in En and Inv neurons suggesting that En/FRU^M^ neurons produce both En and Inv proteins (data not shown).

We further analyzed the En expression pattern in the CNS and other tissues to define the full pattern of En and FRU^M^ co-expression. The neurons expressing *En-GAL4* completely overlapped with the anti-En antibody showing that the driver line recapitulates the normal En pattern in the CNS (data not shown), consistent with other results using the same *En-GAL4* driver and anti-En antibody [46].

Outside the CNS, *En-GAL4* expresses in the En pattern in the posterior epidermis of each body segment, two direct flight muscles, the posterior compartment epithelia of imaginal discs and their adult derivatives, including the genitalia, and sensory neurons derived from the epithelia of the antenna, legs, and genitalia (cf. data not shown). Peripheral sensory neurons in a variety of imaginal derivatives express FRU^M^ proteins at least transiently [15-17,27,28,47]. To determine whether we could find peripheral neurons that co-expressed FRU^M^ and En, we examined external tissues, including genitalia, legs, proboscis, antenna, abdominal body wall and thoracices, from pupal and adult *En-GAL4-membrane GFP* animals that were labeled with both anti-En and FRU^M^ antibodies. We did not find co-expressing sensory neurons at the stages we examined (data not shown). Recent studies have shown that En is expressed in the anterior lobe of the male genital disc that contributes to the development of the internal genitalia and is not expressed in the parts of the disc that contribute to the male external genitalia. Instead, *cubitus interruptus* is expressed in the region from which the genital arch, lateral plate, clasper and hypandrium are derived and from which the FRU^M^-positive sensory neurons are produced [48]. Thus no co-expression of FRU^M^ and En is detected outside of the CNS.

### Expression of *fru*^*M*^-RNAi selectively depletes FRU^M^ within En/FRU^M^ neurons

**Figure 2:**
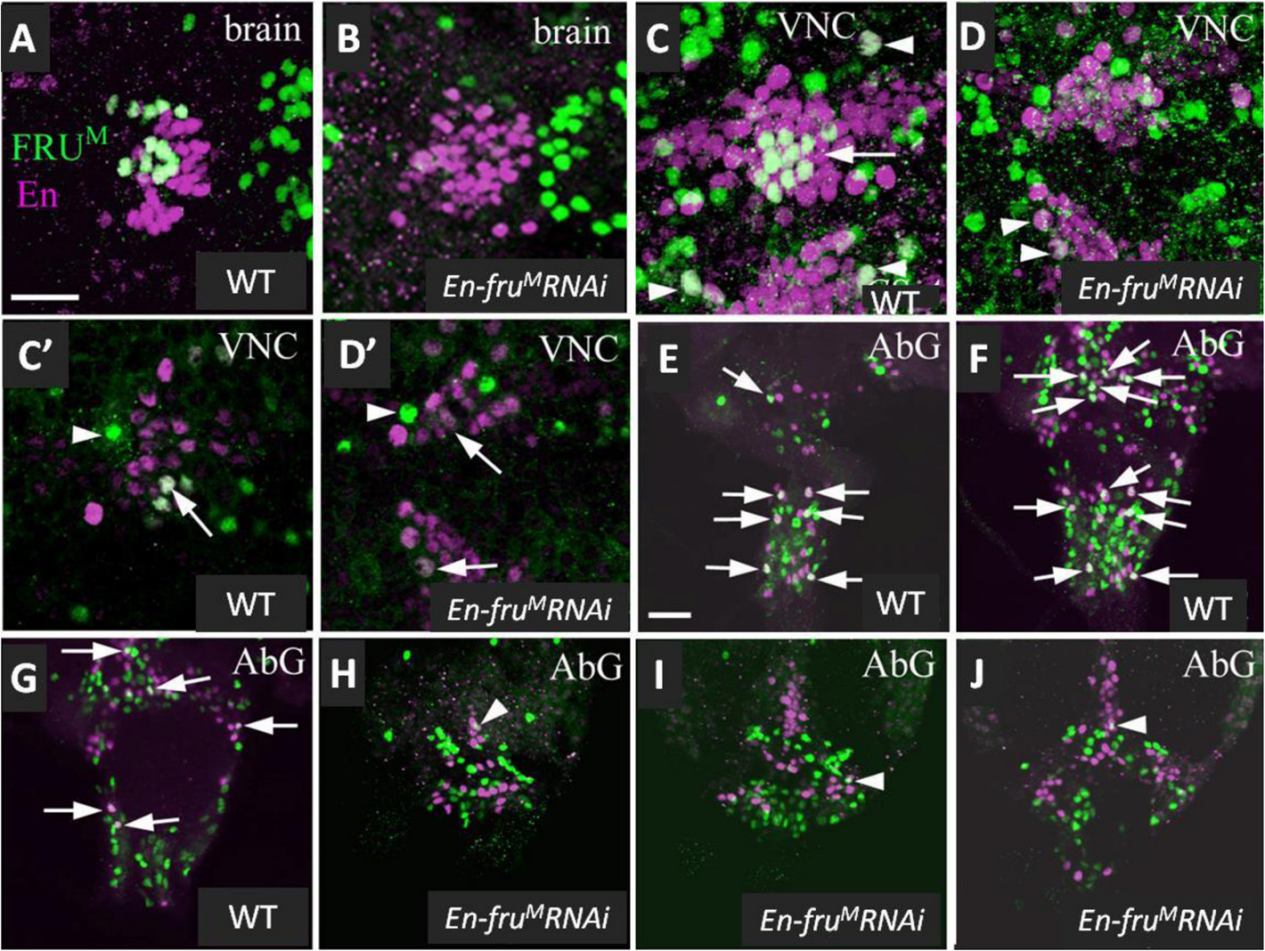
**FRU**^**M **^**is significantly reduced in *En-fru***^***M***^***RNAi *males.** CNSs of wild-type (**A**, **C**, **C’ E**-**G**) and *En-fru*^*M*^*RNAi* (**B**, **D**, **D’**, **H**-**J**) males labeled for anti-FRU^M^ (green) and anti-En (magenta) expression. (**A**) FRU^M^ expression in E/F-brain neurons. (**B**) FRU^M^ is extremely reduced in the E/F-brain neurons of *En-fru*^*M*^*RNAi* males. In the brain, FRU^M^ is reduced by 86% (n = 3, compare **A** to **B**, see results for methods). (**C**) FRU^M^ expression in E/F-VNC_mid_ T1 (arrow) and E/F-VNC_med_ T1 and T2 (arrowheads) neurons. (**D**) FRU^M^ is reduced by 81% in midline (n = 3) and by 77% in medial (n = 3, arrowheads) neurons in *En-fru*^*M*^*RNAi* males. (**E**-**G**) FRU^M^ expression in ventral (**E**), ventromedial (**F**) and medial (**G**) AbG neurons (arrows). (H-I) FRU^M^ is reduced by 77% in AbG neurons (n = 3) of *En-fru*^*M*^*RNAi* males. To clearly show that FRU^M^ has been reduced in all AbG neurons, panels **E**, **F**, and **G** are at three different z-axis positions of the AbG in a WT male and panels H, I, and J are at similar z-axis positions in an *En-fru*^*M*^*RNAi* male. Arrowheads in panels H-J denote neurons that appear to co-express En/FRU^M^ (but do not) because of overlap of two separate neurons in adjacent z-sections. (**C’**-**D’**) Examples of FRU^M^-only reference neurons (arrowheads) and En/FRU^M^ neurons (arrows) are shown in panels C’ (WT male) and D’ (*En-fru*^*M*^*RNAi* male). Images are either confocal z-stacks (**A**-**D**, **E**-**J**) or overlays of single z-sections (**C’, D’**). Size bars = 20 μm (**A**) for panels **A**-**D**, and (**E**) for panels E-J.

Expression of two copies of a *fru*^*M*^-inhibitory RNA (*fru*^*M*^-RNAi) transgene construct (*UAS-fru*^*M*^*IR*[29]) driven by *En-GAL4* was sufficient to reduce FRU^M^ to very low levels in En/FRU^M^ neurons in males raised at 29°C (*En*-*fru*^*M*^-RNAi males, Figure 2). The ratio of anti-FRU^M^ pixel intensity in depleted En/FRU^M^ neurons to that of adjacent control FRU^M^-only reference neurons was used to normalize data for neurons sampled in each group (see Methods). In En/FRU^M^ neurons from wild-type males, the anti-FRU^M^ signal was lower than the reference neuron for all neuronal groups. In *En*-*fru*^*M*^-RNAi males, FRU^M^ expression was significantly reduced in all En/FRU^M^ neurons compared to wild-type levels with decreases of 86% in E/F-brain, 81% in E/F-VNC_mid_, 77% in both E/F-VNC_med_ and E/F-AbG (Figure 2). This reduction was significant for neurons in all regions (p < 0.001 for E/F-brain and all E/F-VNC neurons, p < 0.005 for E/F-AbG neurons analyzed by paired t-tests after arcsine transformation of the ratios). En expression begins prior to FRU^M^ expression in all En/FRU^M^ groups. We measured a significant reduction in FRU^M^ levels in 2–3 day old *En*-*fru*^*M*^-RNAi males suggesting that in depleted adults, the manipulated En/FRU^M^ neurons developed and functioned with only about 14-20% of normal FRU^M^ levels.

### En/FRU^M^ neurons function in male fertility

Fewer *En*-*fru*^*M*^-RNAi males were fertile when housed with several virgin females for one week at 29°C compared to control males (61%, Table 2). In vials with fertile *En*-*fru*^*M*^-RNAi males, we observed that many progeny were produced suggesting that at least some individual males were able to mate and were apparently as fecund as control males. As a second approach to deplete FRU^M^ levels, we used males expressing *UAS-GAL4* in addition to drive the *UAS-fru*^*M*^-construct, and found that fewer *En*-*fru*^*M*^-RNAi*/UAS-GAL4* males were fertile compared with control males. Males of two of the three *En*-*fru*^*M*^-RNAi*/UAS-GAL4* lines were additionally less fertile than *En*-*fru*^*M*^-RNAi males (Table 2). To assess the relationship between mating frequencies in single pair tests to fertility results from week-long tests, twenty-three individual *En*-*fru*^*M*^-RNAi males were transferred directly from the 10-minute courtship assay to food vials with 2–3 virgin females for a one-week general fertility test. Only fourteen of these males (61%) were fertile. The percentages of fertile *En*-*fru*^*M*^-RNAi and control males from this experiment were the same as that found in the original one-week fertility tests. Failures in courtship and copulation account for most of the sterile phenotypes of *fru* mutant males, however, fertile matings do occur in certain hypomorphic genotypes associated with copulation abnormalities [6,30,31,49].

**Table 2:** **Mating, copulation duration and fertility phenotypes of FRU**^**M**^**-depleted males**

**Male genotype**	**Percent mated (%)**	**Mean copulation duration ± SEM**	**Percent fertile (%)**
WT	67 (n = 19)	15.6 ±1.0 (n = 19)	88 (n = 16)
*En-GAL4*/+	85 (n = 20)	15.3 ± 0.53 (n = 20)	84 (n = 16)
*fru*^*M*^*RNAi/+*, *UAS-GAL4-I* /+	95 (n = 20)	16.7 ± 1.0 (n = 20)	90 (n = 20)
*En-fru*^*M*^*RNAi*	57 (n = 28)	10.9 ± 1.5 (n = 13)	61 (n = 23)
*En-fru*^*M*^*RNAi*, *UAS-GAL4-I*	19 (n = 21)	11.3 ± 4.4 (n = 21)	25 (n = 20)
*En-fru*^*M*^*RNAi*, *UAS-GAL4-II*	35 (n = 20)	12.9 ± 2.2 (n = 20)	40 (n = 20)
*En-fru*^*M*^*RNAi*, *UAS-GAL4-III*	55 (n = 20)	13.0 ±1.6 (n = 20)	67 (n = 21)
*En-fru*^*M*^*RNAi*, *Cha-GAL80*	30 (n = 20)	19.0 ± 3.9 (n = 20)	42 (n = 19)

All males were raised and maintained at 29°C. Single males (n = number tested) and females were paired for 30 minutes and the percent mated and the mean copulation duration calculated in minutes. Fertility tests were carried out at 29°C between an individual male and 2–3 virgin WT females for 7 days and the presence of larvae determined. For these experiments, the *En-GAL4* chromosome had been backcrossed into a *w*^*-*^ background for four generations.

### Males with low FRU^M^ levels in En/FRU^M^ neurons have normal courtship but fail to copulate

**Figure 3:**
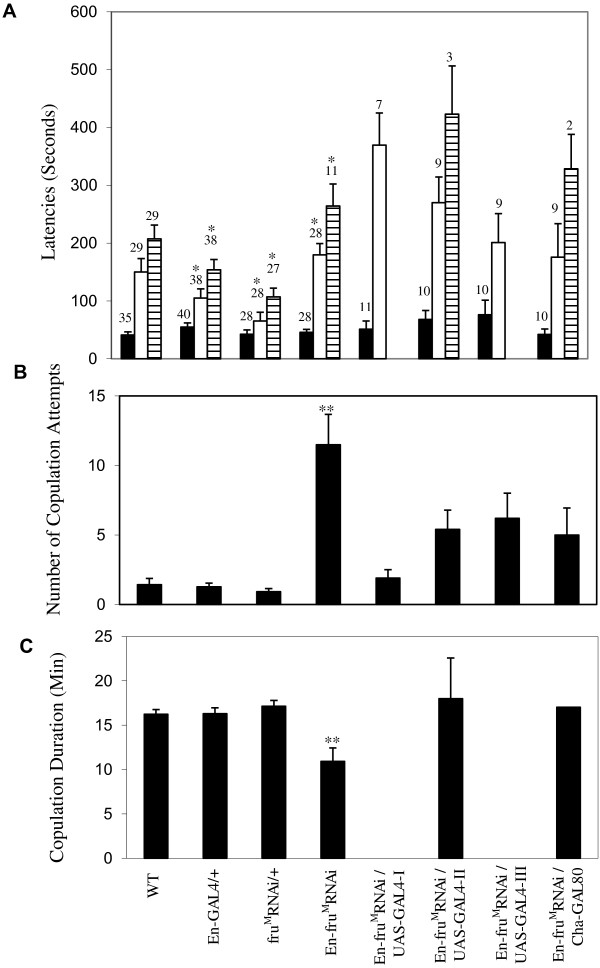
**Courtship and copulation phenotypes of males expressing a FRU**^**M **^**inhibitory RNA transgene compared to control males.** Measurements from 10-minute videotaped courtship tests (see Methods) include **A**) Latencies to courtship initiation (black), first attempted copulation (white) and copulation (stippled), **B**) Number of copulation attempts, and **C**) Copulation duration. The number of animals tested (n) is shown above each bar on the graph (**A**) and genotype labels are indicated beneath graph (**C**). For genotypes missing stippled bars in (**A**) or black bars in (**C**), none of those males achieved copulation. *En-fru*^*M*^*RNAi* males’ values for Latency to courtship initiation (**A**-black) are not statistically different from controls (p = 0.265). The average durations for *En-fru*^*M*^*RNAi* males measured as Latency to the first attempted copulation (A-white), Latency to copulation (A-stippled) are significantly different from durations measured for *En-Gal4*/+ and *fru*^*M*^*RNAi/*+ controls (p < 0.001)*. *En-fru*^*M*^*RNAi* males have more copulation attempts (**B**) and shorter copulation durations (**C**), which are significantly different from values for all other genotypes (p < 0.001)**. All data are shown as mean ± SEM.

To test the hypothesis that *En*-*fru*^*M*^-RNAi males produced abnormal courtship, we paired individual males with single females in small courtship chambers (see Methods). These males exhibited the full range of courtship behaviors, including orientation to and following the female, wing extension and vibration, licking, and tapping. By two important criteria, the courtship of *En*-*fru*^*M*^-RNAi males was robust: 1) These mutant males initiated courtship with the same latency as WT and control males (Figure 3A); 2) The courtship index (CI) of *En*-*fru*^*M*^-RNAi males, a measure of the amount of time spent courting, was not significantly different from WT and control males (Additional file 1: Table S1). However, fewer *En*-*fru*^*M*^-RNAi males mated (57%, Table 2). Likewise, fewer *En*-*fru*^*M*^-RNAi*/UAS-GAL4* males mated (19%, 35%, 55% *En*-*fru*^*M*^-RNAi*/UAS-GAL4-I, En-fru*^*M*^*-RNAi/UAS-GAL4-II, En-fru*^*M*^*-RNAi/UAS-GAL4-III*, respectively; Table 2). These *En*-*fru*^*M*^-RNAi males did attempt copulation with an average of 12 unsuccessful copulation attempts with 21% of these males making over 20 attempts. By comparison, WT and control males average 1.5 attempts and 40% of WT and control males were successful on their first attempt (13/35 WT; 17/40 *En-GAL4*/+; 13/38 *UAS-fru*^*M*^*RNAi* /+; Figure 3B). These data confirm that *En*-*fru*^*M*^-RNAi males produce very active courtship and suggest that male sterility is due at least in part to the failure to make the transition from male–female genital contact to a stable copulation stance. Further, these findings demonstrate that the copulation defects in *En*-*fru*^*M*^-RNAi males are due specifically to depletion of FRU^M^ in En neurons and not to genetic background effects. Moreover, these data suggest that further reduction of FRU^M^ leads to more severe, but not additional, copulation phenotypes.

### Expression of FRU^M^ in En neurons is necessary for maintenance of successful copulations

In the course of these courtship/copulation experiments, we discovered that *En*-*fru*^*M*^-RNAi males often had abnormal courtship durations compared to control males (Figure 3C). The average copulation duration for control males was around 16 minutes, consistent with previously published results [30], while *En*-*fru*^*M*^-RNAi males mated with a wide range of copulation durations. The mean duration was11 minutes, which is significantly different from that of controls (Figure 3C).

**Figure 4:**
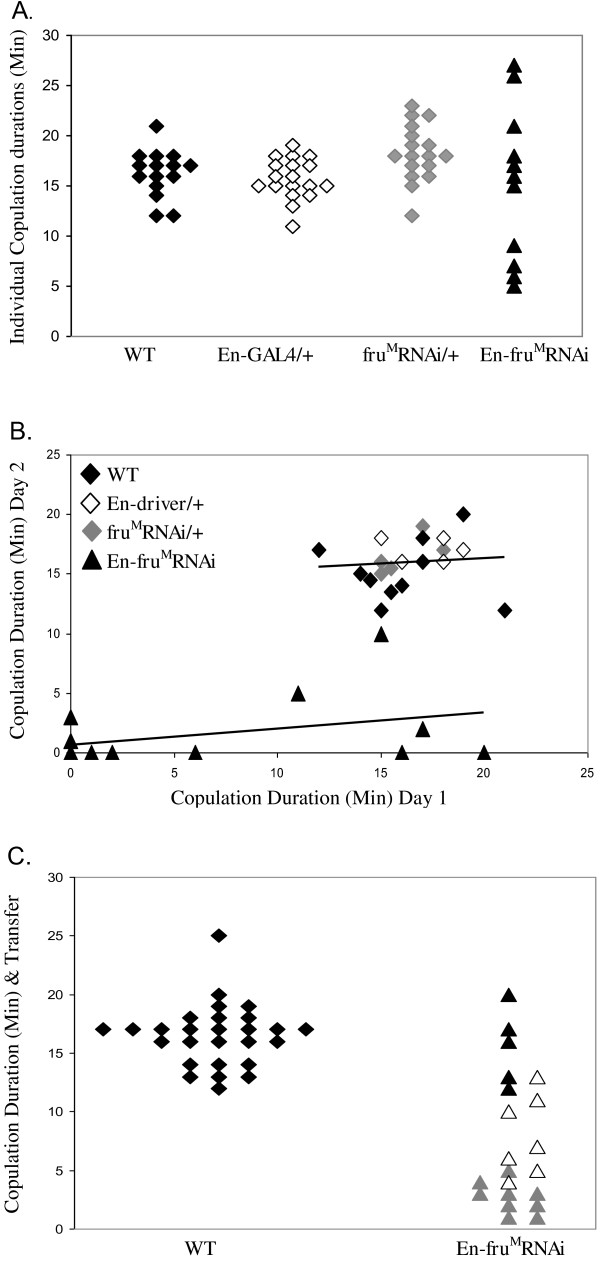
***En-fru***^***M***^***RNAi *males have abnormal copulation durations.***En-fru*^*M*^*RNAi* males’ copulation durations vary and often failed to transfer sperm and mating plug material. (**A**) Scatter plot of individual copulation durations. Mean copulation durations with wild-type females ± SEM are WT, 16.3 ± 0.6 min; En-GAL4/+, 15.7 ± 0.4 min; *fru*^*M*^*RNAi*/+, 18.3 ± 0.7 min; *En-fru*^*M*^*RNAi*, 15.2 ± 2.2 min. (**B**) Individual durations on two consecutive days, each with a fresh wild-type female.  For controls, mean ± SEM duration on day 1 and day 2 was not statistically different between genotypes or days (p = 0.622); WT = 16.2 ± 0.8 min, 15.3 ± 0.8 min; *En-GAL4*/+ = 17.2 ± 0.7 min, 17.0 ± 0.5 min; *fru*^*M*^*RNAi* /+ = 16.0 ± 0.6 min, 16.4 ± 0.7 min.  Durations for *En-fru*^*M*^*RNAi* males were different between day 1 and day 2, 11.0 ± 2.5 min, 4.2 ± 1.6 min; four *En-fru*^*M*^*RNAi* males did not mate on either day. Regression analysis for Day 1 versus Day 2 durations show that Day 1 duration does not predict Day 2 duration: control genotypes grouped, R2 = 0.006, p = 0.744; *En-fru*^*M*^*RNAi* males R2 = 0.137, p = 0.193. (C) Sperm and mating plug transfer was assessed in relation to copulation duration. WT (n = 32) and *En-fru*^*M*^*RNAi* (n = 38) males were used; all males that copulated are shown. Here, 84% of WT males mated with a mean duration of 16.5 ± 0.5 minutes, compared to only 55% of *En-fru*^*M*^*RNAi* males mated, with a mean duration of 7.5 ± 0.9 minutes. Males that transferred sperm and a mating plug are filled symbols (both genotypes). In the case of *En-fru*^*M*^*RNAi* males, those that transferred a mating plug, but no sperm are open symbols and those that did not transfer sperm or a mating plug are gray symbols.

To understand better the copulation phenotypes of *En*-*fru*^*M*^-RNAi males, copulation durations were measured in a separate set of experiments (Figure 4A-C). Control and *En*-*fru*^*M*^-RNAi males were paired individually with mature virgin females, allowed 30 minutes maximum to initiate copulation, and duration determined. Nearly 50% of the *En*-*fru*^*M*^-RNAi males in these experiments failed to mate within the 30 minute period (see Figure 4 legend). For the *En*-*fru*^*M*^-RNAi males that did mate, there was a wide range of copulation durations, from five minutes to 27 minutes, whereas control males had more uniform copulation durations (13–22 minutes, Figure 4A) similar to the copulation durations observed after 10-minute courtship and copulation tests (see Figure 3C).

Because of the variability in the copulation durations measured for *En*-*fru*^*M*^-RNAi males, we determined whether individual males consistently had short duration copulations or repeatedly failed to copulate by measuring the durations of serial copulations for a given male (Figure 4B). Of the fourteen males tested, only three *En*-*fru*^*M*^-RNAi males mated on consecutive days, seven males mated on only one of the two days and four males did not mate either day. By contrast, all control males mated on both days. The mean copulation durations for *En*-*fru*^*M*^-RNAi males were 11.0 ± 2.5 minutes on day 1 and 4.2 ± 1.6 minutes on day 2, significantly shorter than control values on both days, around 16 minutes (Figure 4B). Thus, the copulation phenotype of *En*-*fru*^*M*^-RNAi males shows both variable expressivity and penetrance (Figure 4).

Our data raised the possibility that some *En*-*fru*^*M*^-RNAi males might be sterile due to the failure to copulate long enough to transfer sperm, seminal fluids, or both components to females [50]. To more thoroughly assess the relationship between copulation duration and fertility, females from matings with known copulation durations (Figure 4B) were transferred singly to food vials. Females mated with control males (n = 20) for durations ranging from 12 to 21 minutes produced offspring. Only two matings by control males resulted in females that did not produce offspring, and both of these males had fertile matings on the second day. Of the females that mated with *En*-*fru*^*M*^-RNAi males (Day 1, n = 8; Day 2, n = 4), only two females produced offspring and these mated for 16 and 20 minutes (Figure 4B). When the reproductive tracts of females that mated but did not produce offspring (n = 10) were dissected, no sperm was present (data not shown). Seven of these sterile copulations were shorter than the 7–10 minute period when sperm is normally transferred [50] however sperm was not transferred during some copulations that lasted for 11, 15 or 17 minutes, well within the range of successful copulation durations of control males.

To determine the timing for transfer of components during copulation, reproductive tracts were removed within 60 minutes after copulation and viewed under fluorescent optics to visualize mating plug material and by differential interference contrast (DIC) optics to visualize sperm (Figure 4C). Control males had copulation durations of 12 to 25 minutes and always transferred both the sperm plug and sperm to the female. *En*-*fru*^*M*^-RNAi males that mated with copulations of three minutes or less did not transfer either sperm or sperm plug material, males that mated with copulation durations between 4–11 minutes only transferred sperm plug material and males that mated with copulations lasting at least 13 minutes transferred both sperm and sperm plug material. These times are in general agreement with the timeline of when components are transferred during wild-type copulations [50].

Examining the data regarding both the fertility and the transfer of sperm and seminal fluids, 30% of *En*-*fru*^*M*^-RNAi males that mated with wild-type durations (≥12 minutes, n = 10) did not transfer sperm, even though these *En*-*fru*^*M*^-RNAi males manufactured apparently wild-type levels of sperm plug material and motile sperm (Additional file 2: Figure S1). These findings suggest that some copulating *En*-*fru*^*M*^-RNAi males fail or have delays transferring sperm and mating plug material compared with controls, but generally, if copulation lasted long enough, both sperm and sperm plug material could be transferred by these males. We find that although *En*-*fru*^*M*^-RNAi males make normal levels of sperm and accessory fluids, they have several defects associated with achieving a successful/stable copulatory position as well as differences in the timing or failure to transfer sperm and accessory material transfer, resulting in a higher frequency of sterile matings. These data suggest that only a small fraction (23%) of copulations by *En*-*fru*^*M*^-RNAi males would be expected to be fertile. When coupled with the high rate of failed copulation attempts, typically 50-57%, it is surprising that males reach an overall fertility rate of 60 percent (Table 2). We suggest that over the one-week period for the fertility test, males must have copulated at least twice to reach frequency of 61% fertility.

Although there was no overlap of FRU^M^ and En protein expression in the peripheral nervous system, we carried out an additional control to address the possibility that the copulation phenotypes could be due to disruption of peripheral nervous system function. *En*-*fru*^*M*^-RNAi/*Cha-GAL80* males were generated, with the *Cha-GAL80* transgene acting to repress GAL4 activity in cholinergic neurons, including all primary sensory neurons [51]. Thus in *En*-*fru*^*M*^-RNAi /*Cha-GAL80* males, the *FRU*^*M*^*-RNAi* construct is not transcribed in the peripheral sensory neurons, leading to wild-type FRU^M^ levels in peripheral neurons but depleted FRU^M^ in central En/FRU^M^ neurons *En*-*fru*^*M*^-RNAi/,*Cha-GAL80* males were paired individually with wild-type virgin females and examined for courtship and copulation (see Methods). These males did not have courtship defects but did have abnormalities in copulation and fertility, similar to the phenotypes of *En*-*fru*^*M*^-RNAi males (Figure 3, Table 2). This finding indicates that the copulation phenotypes of *En*-*fru*^*M*^-RNAi males are not due to FRU^M^ depletion in the peripheral nervous system but to a defect originating within the CNS neurons described here.

### The copulation defects and sterility in *En*-*fru*^*M*^-RNAi males are not due to abnormal locomotor activity levels or abnormal reproductive structures

Post-mating sterility of certain *fruitless* mutant males has been linked to defects in a group of serotonergic neurons in the dorsal posterior abdominal ganglion, which are either missing or fail to express serotonin in females and *fru* mutants [26,49]. In a wild-type male, the axons of these serotonergic neurons project down the main abdominal nerve to form terminal arborizations on the accessory glands, testicular ducts, seminal vesicles, vas deferens, and anterior ejaculatory duct [26]. To determine whether the sterility of *En*-*fru*^*M*^-RNAi males might be due to loss of this serotonergic innervation, reproductive tracts were labeled with anti-serotonin antibody and serotonergic nerve terminals were present and appeared to be at wild-type levels on the same set of reproductive structures (Additional file 3: Figure S2). Therefore, the defects in copulation in *En*-*fru*^*M*^-RNAi males do not appear to be due to defects in the serotonergic innervation on the male internal reproductive organs.

To rule out the possibility that *En*-*fru*^*M*^-RNAi males had some defect in general activity or a physical defect in sex-specific and/or reproductive structures that might be responsible for the copulation phenotypes, the locomotor activity levels and anatomy of *En*-*fru*^*M*^-RNAi were compared with controls. There were no differences in overall activity of *En*-*fru*^*M*^-RNAi compared to control males measured over a twelve day period (p = 0.214, One-way ANOVA); *En*-*fru*^*M*^-RNAi males (n = 8) made 18.7 ± 1.9 line crossings per half hour, compared to 15.2 ± 1.6 for *En-GAL4/+* (n = 8) and 15.2 ± 1.1 for *fru*^*M*^*RNAi* /+ males (n = 8). In addition, since *En*-*fru*^*M*^-RNAi males perform courtship as robustly as control males as measured by CI (Additional file 1: Table S1), it is unlikely that reduced locomotion accounts for the lower copulation success of *En*-*fru*^*M*^-RNAi males.

Finally, the external and internal genital cuticular structures and abdominal-genital musculature of control and *En*-*fru*^*M*^-RNAi males were examined and no differences were found in the morphology of these structures or in the Muscle of Lawrence (MOL), a male-specific dorsal abdominal muscle that is missing or defective in *fru* mutant males (data not shown).

### Females and *fru* mutant males have homologues to WT male En/FRU^M^ neurons

**Figure 5:**
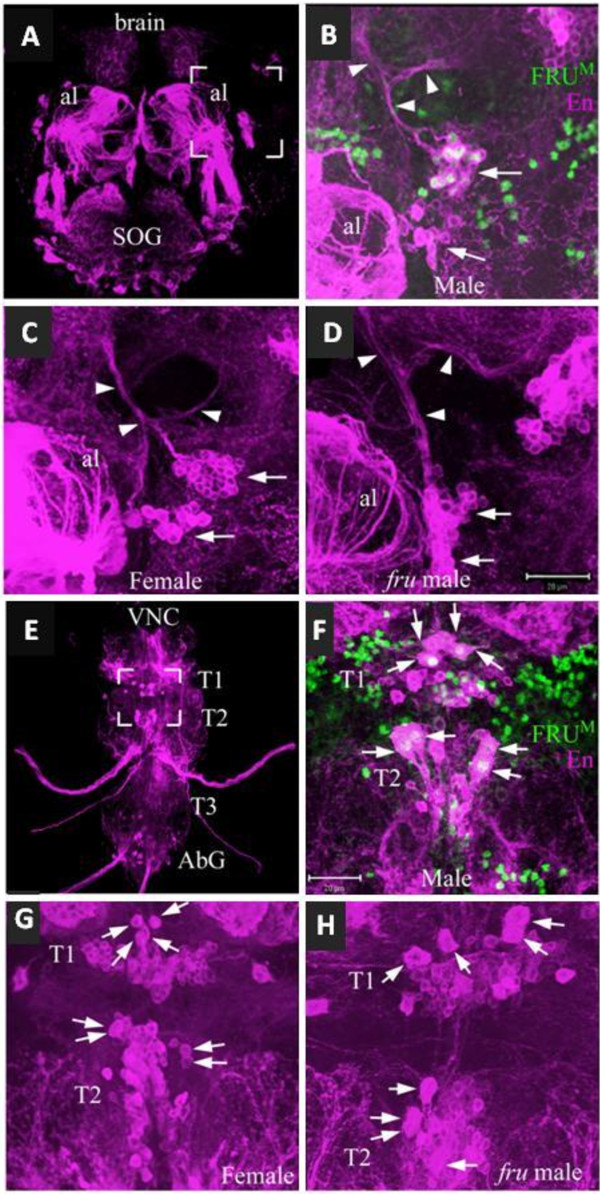
**En/FRU**^**M **^**neurons have the same initial projections in males, females, and *fru*-mutant males.** CNSs of wild-type males (**B**, **F**), wild-type females (**C**, **G**), and *fru*-mutant males (**D**, **H**) were labeled for anti-FRU^M^ (green) and *En-GAL4* expressing membrane-bound GFP (magenta). (**A**, **E**) Engrailed neuronal projections in the brain and VNC of wild-type males. Bracketed regions indicate zoom areas for panels **B**-**D** and **F-H**. (**B**) E/F-brain neurons appear to be in two distinct groups, which project anteriorly, then bifurcate. The same neurons are seen in females (**C**) and *fru*-mutant males (**D**) which show the same projection pattern but do not express FRU^M^. (**F**) E/F-VNC neurons in T1 include the midline group in T1 and the medial groups in T1 and T2. Although the projection patterns are difficult to discern, similar neurons are clearly present in females (**G**) and *fru*-mutant males (**H**). Size bars = 20 μm (**D**) for panels **B**-**D**, and (**F**) for panels **F**-**H**.

Having identified a set of En/FRU^M^ neurons in males, we considered the possibility that these neurons might be present exclusively in males as found for some anterior brain neurons [33] or whether females and *fru* mutants might also have these neurons. We used two approaches to assess the presence of these neurons: 1) we counted the number of En neurons at different developmental stages in males, females and *fru* mutants (Table 3), and 2) we labeled CNSs from *En-GAL4* expressing membrane-bound GFP with anti-FRU^M^ (Figure 5).

**Table 3:** **Number of En cells in brain and thoracic regions of WT and *fru *mutant CNSs**

	**Anterior medial brain**			**First thoracic ganglion (T1)**		
**Genotype**	**28 hr APF**	**50 hr APF**	**Adult**	**28 hr APF**	**50 hr APF**	**Adult**
**WT male**	47.2 ± 2.1	46.2 ± 2.2	48.4 ± 2.0	107.8 ± 3.5	118.8 ± 3.9	110.7 ± 7.1
	(n = 16)	(n = 9)	(n = 14)	(n = 10)	(n = 10)	(n = 10)
**WT female**	48.4 ± 1.3	35.2 ± 3.9^a^	50.7 ± 2.3	111.2 ± 3.2	117.6 ± 3.1	94.5 ± 5.3
	(n = 19)	(n = 10)	(n = 10)	(n = 10)	(n = 10)	(n = 10)
***fru***^***sat15***^***/fru***^***4-40 ***^**male**	44.5 ± 1.6	47.2 ± 2.5	42.2 ± 2.0	132.2 ± 4.9^b^	114.9 ± 3.3	89.3 ± 7.3
	(n = 10)	(n = 6)	(n = 13)	(n = 5)	(n = 10)	(n = 10)
***fru***^***sat15***^***/fru***^***4-40 ***^**female**	47.5 ± 2.5	36.5 ± 5.0	40.8 ± 1.6	121.2 ± 3.2	105.1 ± 3.7	103.0 ± 7.2
	(n = 8)	(n = 4)	(n = 13)	(n = 5)	(n = 7)	(n = 10)

The average number of En-positive neurons (mean ± SEM) found in anterior medial brain and T1 thoracic ganglia in WT and *fru*^*sat15*^*/fru*^*4-40*^ mutant aged pupae and adults (n, refers to the number of brains analyzed with the hemispheres of the brain counted separately). Differences were analyzed by Kruskal-Wallis One-Way ANOVA followed by Dunn’s Method of Multiple Comparison for values within a given region.

^a^ Significantly different from the mean number of 28 hr APF and adult WT male and female brain and 28 hr APF *fru* female medial brain neurons (p < 0.001).

^b^ Significantly different from the mean number of adult WT female and *fru* male T1 neurons (p < 0.001).

We counted the numbers of En-positive neurons labeled by anti-Engrailed antibody in the medial brain and first thoracic segment (T1) of the VNC at several pupal stages and in adults. In both sexes and *fru* mutants, the number of En neurons increases during larval and early pupal stages of development (Table 3). There were no consistent statistical differences in the numbers of En neurons between males and females or between WT and *fru* mutant animals at any developmental stages (Table 3), indicating that FRU^M^ does not affect the number of En-positive neurons. This is consistent with the finding that FRU^M^ expression begins at 8 hr APF in neurons that already express En.

Examination of CNSs from *En-membrane GFP* males labeled with anti-FRU^M^ antibody revealed overlap of Engrailed and FRU^M^ neurons in similar locations and numbers in females and *fru* males as found in wild-type males (Figure 5). We additionally examined the neurochemical profile of the En/FRU^M^ neurons, to look for any difference between sexes or in *fru* mutants. We labeled adult CNSs with antibodies to gamma-Aminobutyric acid (GABA) or Glutamic acid Decarboxylase (GAD), an enzyme used in GABA synthesis. Most Engrailed neurons are GABAergic, including those that co-express FRU^M^ (Additional file 4: Figure S3), even in females and *fru* mutant males. Because some En neurons may also be serotonergic, we also labeled CNSs with serotonin (5HT) or used a GAL4 driver that expresses in Dopa Decarboxylase (Ddc)-positive neurons [51,52]. We found that the E/F-VNC_med_ neurons are labeled with *Ddc-GAL4*-driven expression but not 5HT in wild-type males. In females and *fru* mutant males, the Ddc-GAL4 expression was much weaker and less consistent, noted by the fact that the staining was lighter in these animals and depending on the CNS, not all four of these cells could be observed routinely in each thoracic segment in females and *fru* males (Additional file 4: Figure S3).

In sum, these data confirm that females and *fru* mutant males have the same set of En neurons, which are equivalent to the En/FRU^M^ neurons in males. Thus, the presence of the same cohort of En neurons in females means that the male-specific functions of En/FRU^M^ neurons must be due to their sexually dimorphic differentiation or function as opposed to the absence of these neurons in females.

## Discussion

### *En*-*fru*^*M*^-RNAi males have mutant phenotypes similar to phenotypes of known *fru* mutants

We have shown that FRU^M^ expression in En/FRU^M^ neurons is necessary in order for males to reliably initiate and maintain copulation leading to frequent male sterility. These copulatory phenotypes have also been described for certain hypomorphic *fru* mutants, even though the phenotypes are often more severe than those of *En*-*fru*^*M*^-RNAi males. For example *fru*^*1*^, *fru*^*3*^, and *fru*^*4*^ homozygous mutant males never attempt copulation and also fail to produce, or have abnormalities in, courtship song [53,54]. Some *fru* males, for example *fru*^*1*^ homozygotes, perform male-male courtship, a phenotype that is not present in *En*-*fru*^*M*^-RNAi males. Other *fru* mutant combinations, however, generate males with behavioral phenotypes that are similar to *En*-*fru*^*M*^-RNAi males. Hypomorphic *fru* mutant males that are able to copulate, for example *fru*^*1*^*/fru*^*3*^, have extremely long copulation durations, and often fail to transfer either or both sperm and seminal fluids [26,31]. Interestingly, *fru*^*1*^*/fru*^*3*^ males lack expression of FRU^M^ in about 50% of AbG neurons [49]. *fru* mutant males, including *fru*^*1*^*/fru*^*3*^ males, have defects in terminal serotonergic neurons that project out of the central nervous system [49]. Since these serotonergic neurons in the terminal ganglion do not express En, we did not anticipate inactivation of FRU^M^ in these neurons. As expected *En*-*fru*^*M*^-RNAi males had serotonergic nerve terminals on their reproductive tracts indicating that these neurons were producing their expected neurotransmitter, thus behavioral effects are due to upstream neurons. Although many fewer neurons have lost FRU^M^ expression in *En*-*fru*^*M*^-RNAi males than in, for example, *fru*^*1*^*/fru*^*3*^ males, the similarity in copulation defects suggests that the same neuronal circuits are affected in these genotypes. Thus using a targeted reduction of FRU^M^ in a defined set of neurons, we have been able to identify a small population of En neurons in the CNS that function to provide robustness in the neuronal circuit that mediates a specific subroutine of male copulation. In normal males this is contingent on the production of early courtship behaviors and was inaccessible in *fru* mutants that show no courtship.

### Phenotypes of *En*-*fru*^*M*^-RNAi males are similar to other copulation mutants

Other genes besides *fru* have been implicated in the regulation of male copulation. Mutants that have phenotypes similar to *En*-*fru*^*M*^-RNAi males, in that they alter the duration of copulation, include *stuck* (*sk*, [55]), *coitus interruptus* (*coi*, [55]), *okina* and *fickle* (*fic*, reviewed in [56]), *lingerer* (*lig*; [57]). Recently, males with mutations in the *period* (*per*) and *timeless* (*tim*) genes were also found to have longer than average copulation durations [58]. Male *fic* mutants have phenotypes most similar to *En*-*fru*^*M*^-RNAi males in that they initiate, but do not maintain, copulation [59]. These *fic* males have difficulty in sustained aedeagus extension via the protractor muscles, although these males also have internal structural problems with the apodeme [59]. We detected no anatomical cuticular or muscular defects in *En*-*fru*^*M*^-RNAi males; however, lack of neuronal integration to sustain protractor muscle contraction is a possibility. In a few cases, *En*-*fru*^*M*^-RNAi males were observed to have difficulty disengaging from the female prior to the end of copulation (KLL unpublished observations), a phenotype associated with *sk* and *lig* mutants. Males expressing the effects of hypomorphic *lig* mutations have difficulty terminating copulation, and interestingly, *lig* null males make many attempts but never achieve copulation [57]. *lig* encodes a set of cytoplasmic proteins expressed in cells of the CNS, and so may be expressed in En/FRU^M^ neurons[60]. Thus, *lig* and *fru* may work in at least partially overlapping sets of neurons to regulate copulation.

### En/FRU^M^ neurons functions as interneurons within a copulatory neuronal circuit

En/FRU^M^ neurons are likely to be interneurons, since their processes do not leave the CNS, and based on their position, size, and similarity to En neurons in other insects. These En interneurons could belong to one or more classes of interneurons including 1) sensory interneurons processing incoming primary sensory information, 2) pre-motor interneurons that contribute to the activity of motorneurons affecting copulation behavior, 3) neurosecretory cells that modulate the activity of other neurons via release of neurochemicals, or 4) neurons forming part of a descending control pathway in which higher centers in the CNS influence the function of neurons in the thoracic and abdominal ganglia [52,61-63].

The examination of En neurons throughout development and the timing of FRU^M^ expression suggests that most of the FRU^M^/En neurons are born post-embryonically, as opposed to embryonic neurons that persist into adulthood and are remodeled for adult-specific functions. Based on the small numbers of embryonic En neurons in the brain, the increase in neurons added during larval and pupal development, and their small size, the E/F-brain and E/F-VNC_mid_ are very likely post-embryonic. With these properties, we speculate that these neurons function as local circuit interneurons within a single ganglion. E/F-brain neurons likely belong to the MC2 lineage described by Kumar et al. [64] based on their location and projection pattern. E/F-VNC_mid_ are almost surely progeny of the median neuroblast, lineage 0 [65], *Drosophila* homologues of neurons identified in the grasshopper as neuronal progeny of the median neuroblast. These neurons express En, use the neurotransmitter gamma-aminobutyric acid (GABA), and are spiking local-circuit interneurons [52,61-63] . At the stages for which we could detect FRU^M^ expression, these midline clusters are part of much larger En groups, approximately 120 neurons in T1, representing multiple *en*-positive lineages [61].

We expect that the local circuit interneurons in the prothoracic ganglion coordinate the movement of the legs and/or wings and that the local circuit interneurons in the abdominal ganglion mediate bending of the abdomen and/or activity of the reproductive organs. A previous study of *Fru*^*M*^*-teashirt* co-expressing local circuit interneurons suggested that these neurons were the primary source of FRU^M^ function in directing courtship song phenotypes [66]. Because the latency to the first copulation attempt were not different between the *En*-*fru*^*M*^-RNAi and control males, it does not appear that the thoracic En/FRU^M^ neurons are necessary components of the *FRU*^*M*^*-teashirt* courtship song neuronal circuit. The En/FRU^M^ neurons in the brain may not be directly involved in copulatory behavior since they are found in regions deemed necessary for male courtship but not copulation behavior [15,16], however we cannot rule out an indirect role in copulation behavior for E/F-brain neurons.

Based on the larger size of E/F-VNC_med_ neurons and the presence of few post-embryonic neuroblasts, these and E/F-AbG may be embryonic. These larger, E/F-VNC_med_ neurons may be part of interganglionic interneuronal circuits [63]. As interganglionic neurons, we speculate that these neurons may have a role in coordination of local neuronal circuits, such as facilitating the movement of legs and abdomen for successful copulation [52,63,64]. Given these two different types of interneurons (local circuit and interganglionic), FRU^M^ may have different roles in the sex-specific differentiation in these two classes of neurons. From our studies, we speculate that the loss of FRU^M^ function in these larger interganglionic neurons maybe the reason that *En*-*fru*^*M*^-RNAi males are less successful at copulation and have reduced fertility.

## Conclusions

We have identified a small subset of FRU^M^ neurons distributed in the brain and ventral cord of males by their co-expression with in En neurons. FRU^M^ expression begins during early to mid-pupal period in neurons already expressing En. Most En/FRU^M^ neurons have a distinctive segmental pattern and contribute to only a part of an En neuronal lineage in the prothoracic midline, anterior medial brain or abdominal ganglia. Only in the four large medial neurons is FRU^M^ expressed in the T1, T2, and T3 segmental homologs. Our data further suggests that the En/FRU^M^ neurons are not unique to males but present in females and *fru* mutant males. The En/FRU^M^ neurons in males are GABAergic or show *Ddc-GAL4* expression indicating that for these characteristics they do not differ from other En neurons in males or females. Based on these observations, we suggest that FRU^M^ likely functions in some aspect of sex-specific differentiation of these neurons, perhaps in their physiology or distal projections, that makes them different than other neurons in the lineage in males and the homologs in females.

Our data strongly support a model in which different groups of FRU^M^ neurons regulate different aspects of courtship and copulation behavior [1,3,4]. In such a model, the nervous system is modular, with designated clusters of interconnected neurons responsible for particular behavioral outputs. It is interesting to speculate what behavioral functions might be served by the En/FRU^M^ neurons in females or the consequence of mis-expression of FRU^M^ in these neurons. Female flies in which FRU^M^ is expressed in all of the *fru*-positive neurons have been shown to produce male courtship behavior but do not have male-like attempted copulation or copulation, perhaps a function of the different size and shape of the abdomen [13,15]. However, it is possible that these neurons are involved in circuits responsible for female reproductive functions since females also coordinate walking movements with mating and egg-deposition.

Our findings show that the loss of FRU^M^ expression in the small cohort of En/FRU^M^ neurons results in a high frequency of male sterility. Because some *En*-*fru*^*M*^-RNAi males are fertile, have apparently normal levels of sperm and capable of transferring sperm and sperm plug material to females, male sterility is not likely due to insufficient sperm. Instead, *En*-*fru*^*M*^-RNAi male sterility is accounted for by a reduced frequency of copulation and/or the failure to adequately transfer sperm and seminal fluids during copulation. Our data from the dual mating experiments also shows that individual males may have a successful copulation and unsuccessful copulation attempts. The lower frequency with which *En*-*fru*^*M*^-RNAi males were able to successfully mate and be fertile has two potential explanations. One, FRU^M^ activity in these En/FRU^M^ neurons is necessary for fine-tuning the neuronal circuit responsible for copulation and, in its absence, the neuronal circuit has a much lower success rate. Two, residual expression of FRU^M^ in these En/FRU^M^ neurons permits the neuronal circuit to work well enough for some successful copulations to occur. We cannot rule out the second possibility. It is possible that additional experiments expressing *UAS-dicer* in conjunction with RNAi might lead to a complete loss of FRU^M^ function in these En/FRU^M^ neurons leading to complete male sterility. Our data suggest that the role of FRU^M^ in these neurons is to shift their differentiation to a male-specific fate. Additional studies, at the individual-cell level, will be important to elucidate how the ability to perform courtship and copulation is built into the nervous system during development and how such circuits are maintained and function in the adult fly.

## Methods

### Fly stocks and crosses

Fly stocks were reared in a 12h: 12h light: dark (12h L/D) cycle at 25°C on standard dextrose medium supplemented with 0.1% Nipagen (p-hydroxybenzoic acid methyl ester; Sigma, St. Louis MO) to inhibit mold. The Canton-S strain, *CS-A* (from Jeffrey Hall, Brandeis University, Waltham, MA), was the source of wild-type (WT) males and females. To create different *fru* mutants, we used *Df(3R)fru*^*4-40*^*(fru*^*4-40*^*),* from which full length non-sexspecific FRU transcripts, encoded by the P3 and P4 promoters, but no sex-specific transcripts are made [30]; *Df(3R)fru*^*sat15*^*(fru*^*sat15*^*),* from which *fru* protein coding sequences are deleted so no *fru* transcripts are made [11]; and *fruP1-GAL4*, in which the GAL4 protein coding region is inserted directly downstream of the P1 translational start site, thus blocking the production of FRU^M^ proteins [15]. For FRU^M^ depletion experiments, we used a strain carrying two RNA mediated interference transgenes, UAS*-fru*^*M*^*IR/CyO;* UAS*-fru*^*M*^*IR*, which targets the 5′ coding sequences of male-specific *fru* transcripts [29]. Additional strains for these experiments included three independent recombinant UAS*-GAL4,* UAS*-fru*^*M*^*IR/CyO; UAS-fru*^*M*^*IR*, lines I, II, and III (denoted as UAS-GAL4 I, II, III), and a UAS*-fru*^*M*^*IR, Cha-GAL80* line (from Devanand Manoli (Stanford University, Palo Alto, CA). The Engrailed and Invected expression pattern*,* respectively, was determined using the *en*^*Xho25*^ and *inv*^*Xba21*^ lines ([44]; from Chihiro Hama, RIKEN Center for Developmental Biology, Kobe, Japan). We used an *engrailed-GAL4* line, *en*-*GAL4*e16E *(en-GAL4*; from Andrea Brand, University of Cambridge, Cambridge, United Kingdom) to drive expression of UAS*-GFP-lacZnls* and UAS*-mCD8GFP* reporters (Bloomington Stock Center). For neuronal labeling experiments, we used a Ddc-GAL4 line (from Jay Hirsh, University of Virginia).

### Immunohistochemistry

Central nervous systems (CNSs) from sexed larvae, pupae and adults were processed for immunohistochemistry according to standard techniques [42]. For staged pupae, white pre-pupae (0 hr after puparium formation [APF]) were collected and aged at 25°C. CNSs were dissected in Phosphate Buffered Saline (PBS), fixed in 4% paraformaldehyde, washed in PBS + 0.1% Triton-X (PBS-Tx), blocked in PBS-Tx + 10% normal goat serum (NGS), incubated in primary antibody overnight at 4°C and then in secondary antibody for 2 to 4 hrs at room temperature before mounting. To eliminate cross reactivity, CNSs were processed for antigens detected by anti-mouse antibodies, blocked with anti-mouse FAB fragments (Sigma) for one hour then processed for antigens detected by anti-rat antibodies. We used the following primary antibodies: rat anti-FRU^M^ (1:400, [11,14,31], rabbit anti-β-galactosidase (βgal) (1:10,000, Cappel, Durham, NC); rabbit anti-serotonin (5HT) (1:500, Sigma); mouse anti-aquorea fluorescent protein (AFP) (1:200, Q-Biogene, Inc., Carlsbad, CA); mouse anti-Engrailed/ Invected (mAb 4D9, 1:5 or 1:10, Development Studies Hybridoma Bank, Iowa City, IA; [42]). Secondary antibodies were conjugated to Alexa-488, -555, -594, or −647 fluorophores (Molecular Probes, Eugene OR), or to horseradish peroxidase (HRP, Jackson ImmunoResearch Laboratories, West Grove, PA) for visualization of the color reaction with diaminobenzidine (DAB, Sigma, St Louis, MO). Fluorescently labeled CNSs were mounted in Prolong (Molecular Probes, Eugene OR) and DAB-labeled preparations were dehydrated in alcohol and mounted in Permount (Sigma, St Louis, MO).

### Imaging and image analysis

Confocal images were obtained on a Zeiss 510-Meta confocal scanning microscope while DIC images were captured from an Olympus Vanox-TX microscope with a Sony DKC-5000 digital camera. Images were subsequently processed for contrast using PhotoShop 5.0.2 (Adobe Systems Inc., San Jose, CA).

### Neuronal analysis

The number of En neurons was counted in anti-En labeled CNSs visualized by DAB and analyzed by Kruskal-Wallis One-Way Analysis of Variance (ANOVA) followed by Multiple Comparison Procedures, Dunn’s Method (SigmaStat, version 2.03, SPSS Inc., Chicago, IL). Fluorescently labeled neurons were counted from stacks of confocal images.

We assessed the anti-FRU^M^ signal in En/FRU^M^ neurons to gauge the level of FRU^M^ depletion in *En-fru*^*M*^*-*RNAi adult male CNSs. In single confocal sections, we assigned a pixel intensity value (ImagePro, Media Cybernetics) to the FRU^M^ signal in En/FRU^M^ neurons and a neighboring, distinct FRU^M^-only neuron, present in the same section, and recognizable in all preparations; the ratio of these values gives a normalized pixel intensity measurement for each En/FRU^M^ neuron. The average FRU^M^ signal was determined for four different neuronal En/FRU^M^ groups in 2 or 3 day old WT (n = 3) and *En-fru*^*M*^*-*RNAi (n = 3) adult male CNSs and analyzed using paired t-tests after arcsine transformation of the ratios (SigmaStat, Version 2.03, SPSS Inc., Chicago, IL).

### Fertility assays

Single virgin males aged for 3–7 days or males used in courtship tests were placed with 2–4 virgin CSA females. Fertility was scored by the presence of progeny after seven days.

### Courtship assays and data analysis

Males for the behavioral tests were reared at 29°C on a 12h L/D cycle, collected within 24 hours of eclosion and aged singly at 29°C for 4–6 days to promote the most effective expression of the RNAi transgene [29]. The *En-fru*^*M*^*-*RNAi males were generated from UAS*-fru*^*M*^*IR/*CyO*;* UAS*-fru*^*M*^*IR* females crossed to *en-GAL4/*CyO males. For control males, wild-type females were crossed to *en-GAL4* males, to UAS*-fru*^*M*^*IR*/CyO; UAS*-fru*^*M*^*IR* males or to wild-type males. Virgin CSA female flies were reared at 25°C on a 12h L/D cycle, collected under light CO_2_ anesthesia within 12 hours of eclosion and aged for 3–5 days *en masse*.

Courtship assays were performed between 6 to 10 hours after lights on. A female and then a male fly were aspirated into a courtship chamber (1.0 cm diameter × 0.5 cm high) the pair was video-recorded until copulation occurred or for 10 minutes. We measured the latency to courtship initiation (lat Court_in_) as the interval after adding the male to his first wing extension. After courtship initiation, the percent of time the male courts is defined as the courtship index (CI; cf[67] ). For males who initiated courtship, the CI was measured for the entire interval between courtship initiation and copulation if the period was less than three minutes, for a three-minute interval prior to copulation or for the last three minutes of the recording period, if males did not copulate. The latency to first attempted copulation (lat 1^st^ Cop_att_) is the interval between courtship initiation and the first instance of genital-genital contact. The number of attempted copulations (# Cop_att_) was counted from the onset of courtship through the entire recording period, or until copulation occurred. The latency to copulation (lat Cop) was measured from courtship initiation until the flies achieved copulation. Before statistical analysis, the raw data for lat Court_in_ and lat 1^st^ Cop_att_ were transformed to the square root of the data and CI and # Cop_att_ were transformed to rank order since these were not normally distributed. The differences between genotypes were analyzed using a Kruskal-Wallis One-Way ANOVA with the source of significant difference determined with Tukey Test for multiple comparisons (Tukey Test; SigmaStat, version 2.03, SPSS Inc.).

### Copulation assays

For some experiments, copulation durations were determined for pairs video recorded for 30 minutes. Statistical comparisons of copulation duration intervals were carried out with a Kruskal-Wallis One-Way ANOVA on Ranks, followed by All Pairwise Multiple Comparison Procedures (Dunn’s Method). After copulation, female reproductive tracts were dissected within 60 minutes of copulation and examined for the presence of sperm and mating plug [31,50].

### Activity assays

General locomotion was quantified in a TriKinetic DAMSystem *Drosophila* Activity Monitor (TriKinetics, Inc., Waltham, MA). Individual males were loaded into single capped tubes and the number of midline crossings was recorded in 30-minute intervals for 12 days at 29°C.

## Abbreviations

5HT: Serotonin; AbG: Abdominal ganglion; APF: After puparium formation; CI: Courtship index; CNS: Central nervous system; Cop: Copulation; CSA: Canton-S strain A; Ddc: Dopa Decarboxylase; En: Engrailed; en-GAL4: *Engrailed-GAL4* e16E insert; E/F-brain: En/FRU^M^ co-expression neurons in the medial brain Engrailed groups; E/F-VNCmid: En/FRU^M^ co-expression neurons in the midline first thoracic ganglion Engrailed group; E/F-VNCmed: En/FRU^M^ co-expression neurons in the medial ventral nerve cord Engrailed groups; E/F-AbG: En/FRU^M^ co-expression neurons in the abdominal ganglion Engrailed groups; FRUM: Male-specific products of the *fruitless* gene; En/FRUM: Neurons expressing both En and FRU^M^ proteins; GABA: Gamma-Aminobutyric acid; GAD: Glutamic acid Decarboxylase; Lat: Latency; T1: First thoracic ganglion; T2: Second thoracic ganglion; T3: Third thoracic ganglion; SOG: Subesophageal ganglion; UASGAL4-I -II, -III: Flies produced using *UAS-GAL4; UAS-fru*^*M*^*IR/CyO*; *UAS- fru*^*M*^*IR* parent; VNC: Ventral nerve cord; WT: Wild-type.

## Competing interest

We have no conflicts of interest in presenting this manuscript.

## Authors’ contributions

KLL designed the study, carried out the fly breeding, immunohistochemistry, microscopy and image analysis, behavioral assays, data analysis, and drafted the manuscript. YSL carried out additional immunohistochemistry and behavioral assays. BJT participated in study design and coordination, and helped to draft the manuscript. All authors read and approved the final manuscript.

## Supplementary Material

Additional file 1: Table S1 *En-fru*^*M*^*RNAi* males have normal courtship index (CI) values. Measurements from 10-minute videotaped courtship tests (see Methods) include courtship index (a measure of time spent performing wing courtship song). All genotypes were not statistically different for courtship index (One-Way ANOVA, p = 0.019). Click here for file

Additional file 2: Figure S1 *En-fru*^*M*^*RNAi* males make and store sperm and mating plug material. In dissected reproductive tracts from wild-type (A, B) and *En-*fru^M^RNAi (C, D) males, sperm was viewed by differential interference contrast microscopy and mating plug material was visible under ultraviolet light. Sperm and mating plug material levels appeared to be normal, and sperm were motile. Click here for file

Additional file 3: Figure S2 *En-fru*^*M*^*RNAi* males have normal serotonergic innervation. Serotonergic nerve terminals innervating the internal reproductive organs were examined in wild-type and *en-GAL4/UAS-fru*^*M*^*IR* males by immunohistochemistry with anti-serotonin (5HT). A) In a wild-type male, serotonergic nerve terminals are present on the seminal vesicles (sv), accessory glands (ag) and ejaculatory duct (ed). B) In an *En-fru*^*M*^*RNAi* male, serotonergic terminals are present on the same organs, similar to wild-type males. Images are confocal z-stacks through the male internal reproductive tract. Size bar = 200 um. Click here for file

Additional file 4: Figure S3 Wild-type males, females, and *fru* mutant males have similar neurotransmitter profiles for brain and VNC-T1midline neurons, but vary for VNC-medial neurons in T1, T2, and T3. CNSs of wild-type males (A, C, F, G, H), wild-type females (B, D, I), and *fru*-mutant males (E, J) were labeled for anti-FRU^M^ (green) and anti-Engrailed (magenta), and a neurochemical marker (blue). (A, B) E/F-VNC_mid_ neurons in males, and the equivalent in females, express gamma-Aminobutyric acid (GABA) neurotransmitter as labeled by anti-GABA antibody. (C, D, E) In males, females, and *fru* mutant males E/F-VNC_mid_ neurons are also labeled by anti-GAD antibody (GAD = Glutamic Acid Decarboxylase, an enzyme for GABA synthesis, localized exclusively to GABAergic neurons). (F, G) E/F-brain (F) and E/F-AbG (G) neurons are also labeled by GAD in wild-type males. (H, I, J) Males expressed a *Ddc*-*GAL4;* UAS-*mcd8::GFP* in E/F-VNCmed neurons of T1, T2 (H) and T3 (not shown). Females (I) and *fru*-mutant males (J) express this driver much more faintly, in with variable penetrance in the equivalent neurons. (Ddc = dopa Decarboxylase, an enzyme for serotonin/5HTsynthesis). (K, L, M) Schematic indicating neurotransmitter profile of En/FRU^M^ neurons in the brain (K), T1/T2 segments of the VNC (L), and T3 and AbG (M). Click here for file
