# Supplementary figures and images for: A small cohort of FRUM and Engrailed-expressing neurons mediate successful copulation in Drosophila melanogaster
Source: BMC Neurosci. 2013 May 21;14:57. doi: 10.1186/1471-2202-14-57 (PMC3664081; doi:10.1186/1471-2202-14-57)

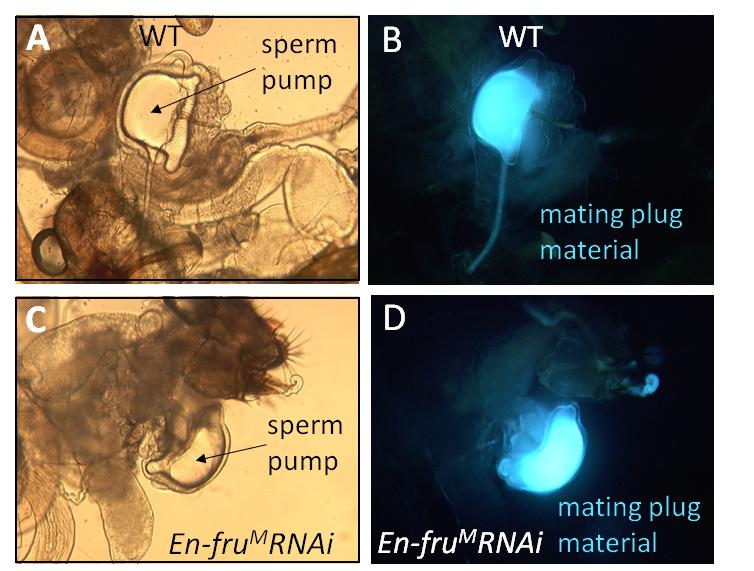

Supplement: Additional file 1: Table S1 — En-fruMRNAi males have normal courtship index (CI) values. Measurements from 10-minute videotaped courtship tests (see Methods) include courtship index (a measure of time spent performing wing courtship song). All genotypes were not statistically different for courtship index (One-Way ANOVA, p = 0.019). [file 1471-2202-14-57-S1.docx]

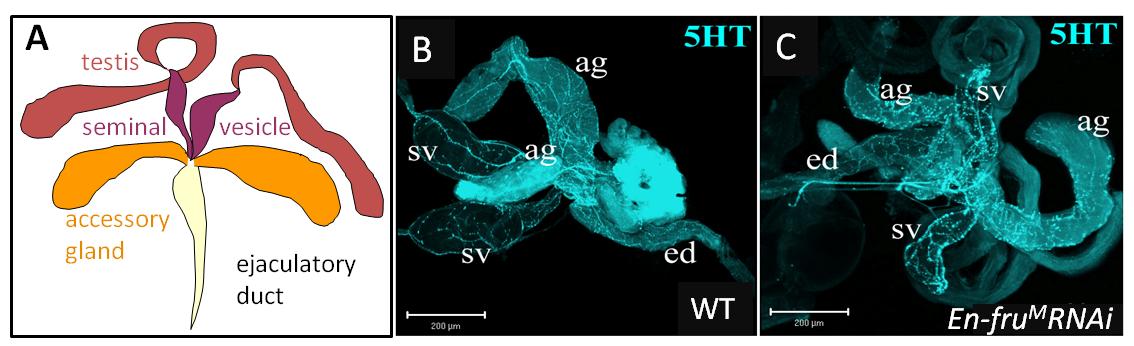

Supplement: Additional file 2: Figure S1 — En-fruMRNAi males make and store sperm and mating plug material. In dissected reproductive tracts from wild-type (A, B) and En-fruMRNAi (C, D) males, sperm was viewed by differential interference contrast microscopy and mating plug material was visible under ultraviolet light. Sperm and mating plug material levels appeared to be normal, and sperm were motile. [file 1471-2202-14-57-S2.docx]

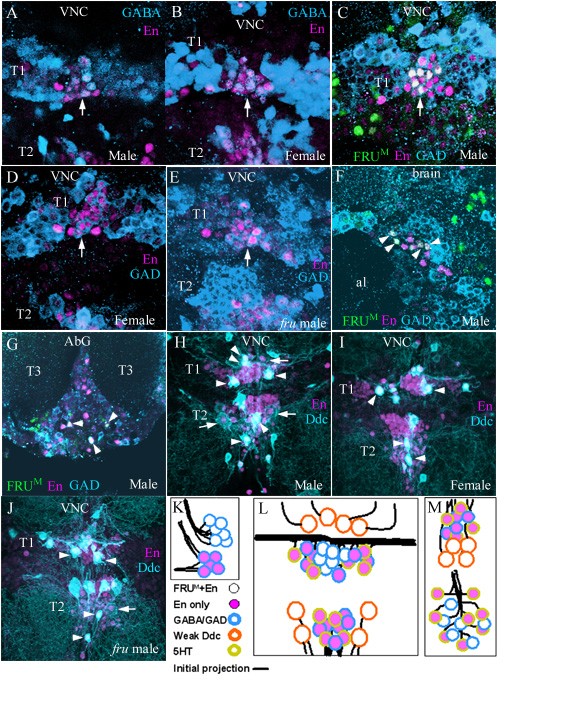

Supplement: Additional file 3: Figure S2 — En-fruMRNAi males have normal serotonergic innervation. Serotonergic nerve terminals innervating the internal reproductive organs were examined in wild-type and en-GAL4/UAS-fruMIR males by immunohistochemistry with anti-serotonin (5HT). A) In a wild-type male, serotonergic nerve terminals are present on the seminal vesicles (sv), accessory glands (ag) and ejaculatory duct (ed). B) In an En-fruMRNAi male, serotonergic terminals are present on the same organs, similar to wild-type males. Images are confocal z-stacks through the male internal reproductive tract. Size bar = 200 um. [file 1471-2202-14-57-S3.docx]
