# Supplementary material for: A small cohort of FRUM and Engrailed-expressing neurons mediate successful copulation in Drosophila melanogaster
Source: BMC Neurosci. 2013 May 21;14:57. doi: 10.1186/1471-2202-14-57 (PMC3664081; doi:10.1186/1471-2202-14-57)
Supplement: Additional file 4: Figure S3 — Wild-type males, females, and fru mutant males have similar neurotransmitter profiles for brain and VNC-T1midline neurons, but vary for VNC-medial neurons in T1, T2, and T3. CNSs of wild-type males (A, C, F, G, H), wild-type females (B, D, I), and fru-mutant males (E, J) were labeled for anti-FRUM (green) and anti-Engrailed (magenta), and a neurochemical marker (blue). (A, B) E/F-VNCmid neurons in males, and the equivalent in females, express gamma-Aminobutyric acid (GABA) neurotransmitter as labeled by anti-GABA antibody. (C, D, E) In males, females, and fru mutant males E/F-VNCmid neurons are also labeled by anti-GAD antibody (GAD = Glutamic Acid Decarboxylase, an enzyme for GABA synthesis, localized exclusively to GABAergic neurons). (F, G) E/F-brain (F) and E/F-AbG (G) neurons are also labeled by GAD in wild-type males. (H, I, J) Males expressed a Ddc-GAL4; UAS-mcd8::GFP in E/F-VNCmed neurons of T1, T2 (H) and T3 (not shown). Females (I) and fru-mutant males (J) express this driver much more faintly, in with variable penetrance in the equivalent neurons. (Ddc = dopa Decarboxylase, an enzyme for serotonin/5HTsynthesis). (K, L, M) Schematic indicating neurotransmitter profile of En/FRUM neurons in the brain (K), T1/T2 segments of the VNC (L), and T3 and AbG (M). [file 1471-2202-14-57-S4.docx]

Supplemental Table 1. ***En-fru^M^RNAi* males have normal courtship index (CI) values.**

|  | **WT** | ***En-GAL4*/+** | ***fru^M^RNAi/+*** | ***En-fru^M^RNAi*** |
| --- | --- | --- | --- | --- |
| Courtship Index (CI) | 83 (n=25) | 76 (n=29) | 76 (n=28) | 88 (n=28) |

Measurements from 10-minute videotaped courtship tests (see Methods) include courtship index (a measure of time spent performing wing courtship song). All genotypes were not statistically different for courtship index (One-Way ANOVA, p=0.019).
